# Starshot field configuration in the linac isocentre size determination: a comparison of film and electronic portal imaging device measurements

**DOI:** 10.2340/1651-226X.2026.45692

**Published:** 2026-06-24

**Authors:** Ari-Pekka Honkanen, Antti Kulmala

**Affiliations:** a Comprehensive Cancer Center, Helsinki University Hospital, Helsinki, Finland; b Clinical Research Institute HUCH Ltd., Helsinki, Finland

**Keywords:** External radiotherapy, quality assurance, winston-lutz, starshot, isocentre

## Introduction

The mechanical precision of the treatment unit is paramount in the successful administration of radiotherapy. This is particularly true for stereotactic radiosurgery where sub-millimetre geometric accuracy is desirable [1, 2]. Periodic quality assurance (QA) is a must to ensure that the unit conforms to the requirements. Vendor-provided machine performance tests can ease the burden of periodic QA, but they do not remove the need for independent verification [3–6].

Two classical methods to assess the mechanical stability of the radiation isocentres of the external photon therapy unit are the starshot and Winston-Lutz tests. In the starshot test, a radiographic film is irradiated with narrow beams at multiple gantry/collimator/couch angles. The stability of the rotation axis is assessed by examining the intersection of the beams [7]. The starshot method can be automated [8], is magnetic resonance imaging (MRI) -compatible and applicable to treatment units without electronic portal imaging devices (EPID). The Winston-Lutz test involves taking portal images of a metal bead positioned at the estimated isocentre and evaluating its imaged position with respect to the radiation field edges as a function of rotation angle [9]. Traditionally the Winston-Lutz test was irradiated onto film but nowadays EPID may be used for a fully digital workflow and to extract additional information from it [10–12].

In this work, we compare an EPID-based Winston-Lutz test using a starshot irradiation pattern with the film-based starshot test in the determination of isocentre size and shape in the transaxial plane. We examine several different treatment units, collimations and beam types.

## Material and methods

Three different treatment units were investigated: a Varian TrueBeam (with a Millenium multileaf collimator [MLC]), an Elekta Versa HD (Agility MLC) and a Varian Halcyon (dual-layer MLC). With the TrueBeam we measured its flattened and flattening filter free 6 MV photon beams (6X-FLT and 6X-FFF, respectively) with jaw and MLC collimations. This specimen was particularly interesting because the focal point of its 6X-FFF beam had a small lateral/transversal misalignment, which results in an approx. 1.0 mm central axis (CAX) deviation of the jaw-collimated crossplane beam profile at the isocentre.

With the Versa HD we measured flattened 6 and 10 MV beams (6X-FLT and 10X-FLT, respectively) collimated with a combination of the MLC and jaws. With the Halcyon the dual-layer MLC collimated flattening filter free 6 MV beam (6X-FFF) was measured. The collimations for these units were dictated by their technical properties as Versa HD has only one set of jaws and Halcyon is jawless.

The film measurements were done using an approx. A4-sized piece of GafChromic EBT4 film sandwiched between two slabs of solid water. The phantom was positioned at the isocentre using the lasers so that the film was in the transaxial plane. The film was irradiated at 15 gantry angles in 24° intervals starting from 186° (in IEC61217 scale). The transaxial width of the fields was nominally 1 cm at the isocentre. Each angle was irradiated twice at the collimator rotations of 90° and 270° with 150 MU each to counteract possible asymmetries in collimation. The film was digitised twice to minimise the errors in the digitisation process. A self-made FORTRAN programme was used to determine the field CAXs on the film. The isocentre was taken to be the point in the middle of the CAX intersection from which the deviations of the individual CAXs were calculated.

The EPID measurements were performed using a Winston-Lutz phantom provided in the Elekta Synergy Basic Calibration Kit MRT 15991. The phantom consisted of a metal bead approx. 8 mm in diameter embedded into a cylindrical acrylic rod. The bead was positioned to the isocentre using kilovolt cone beam computed tomography (CBCT)-imaging. Portal images of the bead were acquired with the EPID using 10 MU square fields at 15 gantry angles in 24° intervals starting from 186°. The nominal field size was 2 × 2 cm at the isocentre. At each gantry angle the images were taken at five collimator rotations divided evenly over the rotational range (TrueBeam and Versa HD: 0°, 72°, 144°, 216°, 288°; Halcyon: 0°, 45°, 90°, 270°, 315°). The portal images were fed into a self-made Python programme, which automatically determines the centre positions of the fields and the bead. Using the bead as a fixed reference point, the CAXs were determined for each gantry angle from the mean of the field centres over the collimator rotations. The isocentre was defined as the point with the smallest sum of the squared distance from it to all the CAXs projected into 3D space. Only the transversal components of the CAXs and isocentre were considered for the further analysis as the longitudinal one has no counterpart in the film measurements. The technical details of the computer programmes are presented in the Supplementary material.

## Results

**Figure 1:**
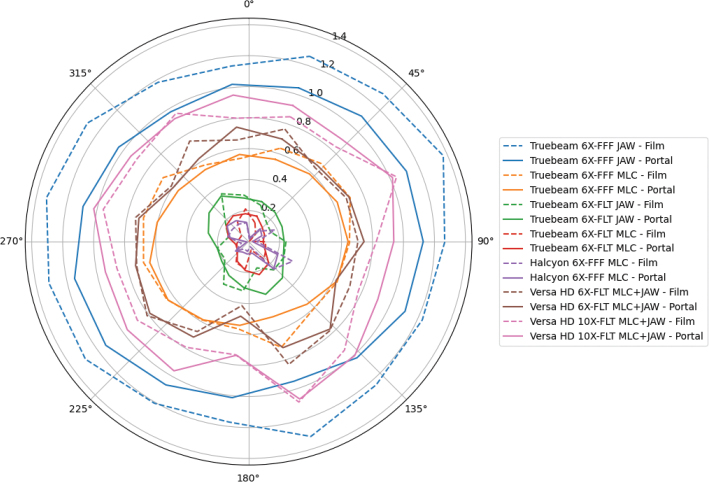
The deviation of the collimator rotation axis/beam central axis from the determined isocentre as a function of the gantry angle. The polar coordinate presented at the outer rim of the plot represents the gantry angle in IEC61217 scale in degrees and the radial coordinate the distance of the axis from the isocentre in mm. The solid lines are the results of the EPID measurements and the dashed lines the film measurements. The different tested beams are identified with colors and they are indicated in the legend on the right. EPID: electronic portal imaging devices.

The measured transversal deviations of CAX from their estimated isocentres are presented in Figure 1. Except for the jaw-collimated 6X-FFF of the TrueBeam, the two methods yield similar deviations for all the tested collimations, beam energies and units. These are quantified in Table 1 where means, standard deviations, and maxima of the differences between the methods are presented. Besides the exception, the mean of differences over all the gantry angles were within 0.08 mm for all the beams and collimations. This is of the same order as their calculated standard errors. The differences between the methods were within 0.20 mm for any particular gantry angle.

**Table 1:** The means, standard deviations and maxima of differences between centre axis deviations from the isocentre between the film and electronic portal imaging measurements over the 15 sampled gantry angles.

Unit, beam, collimation	Mean difference (mm)	Standard deviation (mm)	Maximum difference (mm)
Halcyon 6X-FFF MLC	–0.006 ± 0.011	0.04	0.11
Truebeam 6X-FFF JAW	–0.20 ± 0.02	0.07	0.37
Truebeam 6X-FFF MLC	–0.06 ± 0.02	0.06	0.20
Truebeam 6X-FLT JAW	0.04 ± 0.02	0.07	0.18
Truebeam 6X-FLT MLC	0.041 ± 0.015	0.06	0.17
Versa HD 6X-FLT MLC+JAW	–0.01 ± 0.02	0.06	0.13
Versa HD 10X-FLT MLC+JAW	0.07 ± 0.02	0.06	0.17

The exception to the trend, the jaw-collimated 6X-FFF of the TrueBeam, exhibits systematically larger CAX deviations (0.2 mm on average) in the film measurement than in the EPID one. This is reflected in the larger maximum difference of 0.37 mm, but the standard deviation of 0.07 mm is in line with the other measurements. The MLC-collimated 6X-FFF exhibits no such systematic difference between the methods. However, compared to the 6X-FLT beams of the TrueBeam, the CAX deviations are clearly larger for 6X-FFF: the mean deviations were 1.1–1.3 mm for the jaw-collimated 6X-FFF and 0.6 mm for the MLC-collimated 6X-FFF in comparison to 0.2–0.3 mm and 0.1–0.2 mm deviations of the jaw- and MLC-collimated 6X-FLT beams, respectively.

## Discussion and conclusion

The measured transversal CAX deviations from the estimated isocentre between the film-based starshot and EPID-based Winston-Lutz methods were found to agree within 0.2 mm for every gantry angle for all but one beam. This is in accordance with the previously reported accuracy of the Winston-Lutz test with a similar number of sampled gantry angles [10].

The observed 0.2 mm systematic difference between the methods in the jaw-collimated 6X-FFF of the TrueBeam is most likely owing to the differences in the collimation and the resulting geometric magnification of the focal spot misalignment. In the film test the beams were collimated with the proximal jaws only whereas in the EPID measurements both proximal and distal jaws were used. Using basic geometry, a focal spot shift is magnified at the isocentre by a factor of , where SAD is the source-axis distance and *d* is the distance of the midpoint of the collimation device from the source. For TrueBeam SAD = 100 cm and midpoint distances of the proximal jaws, distal jaws and the MLC are 31.9, 40.6, and 50.9 cm, respectively. Therefore, the geometric magnification factors for the proximal jaws, (mean of) proximal+distal jaws, and MLC are 2.13, 1.80, 0.96, respectively. Assuming a focal spot shift of 0.6 mm, this would lead to the respective CAX deviations of 1.3, 1.1, and 0.6 mm projected at the isocentre, which are in accordance with the measured values.

The results indicate that both tests can accurately determine the CAX deviations of a treatment unit. However, the choice of collimation can affect to the results of periodic QA and possibly to the frequency of the service actions triggered by them. If the MLC is the primary mode of collimation, then performing QA using jaw-collimated beams can lead to an overly pessimistic estimation of the clinical performance of the unit and cause unnecessary maintenance and downtime. On the other hand, the observed differences between the MLC- and jaw-collimated beams can shed light on the nature of the CAX deviations and thus aid in their troubleshooting.

In conclusion, we have compared an EPID-based Winston-Lutz test using a starshot irradiation pattern to a film-based starshot test in the determination of the isocentre size and shape of external radiotherapy units. In terms of the measured transversal CAX deviations from the estimated isocentre, the methods were found to agree within 0.2 mm in the typical case. However, systematic differences were observed in the presence of the focal spot misalignment. These are most likely attributed to differing geometric magnifications at the isocentre which depend on the used collimation device. This may impact the interpretation of QA results and maintenance actions taken based on them.

## Supplementary Material



## Data Availability

The data are available from the authors upon a reasonable request.
