# Supplementary material for: Starshot field configuration in the linac isocentre size determination: a comparison of film and electronic portal imaging device measurements
Source: Acta Oncol. 2026 Jun 24;65:45692. doi: 10.2340/1651-226X.2026.45692 (PMC13309924; doi:10.2340/1651-226X.2026.45692)
Supplement: Supplementary file 1 [file AO-65-45692-s1.pdf]

Supplementary material has been published as submitted. It has not been copyedited, or typeset by Acta Oncologica

Starshot test in the linac isocentre size determination – comparison of film and electronic portal imaging device measurements: Supplementary material

Ari-Pekka Honkanen and Antti Kulmala

Technical description of the portal image analysis

The location and the size of the isocentre is determined from the images of a bead phantom taken with an electronic portal imaging device (EPID) as follows. Firstly, the centres of the fields are located based on the centre point of the fitted quadrilateral to the recognised field edge points. Then the field centres are used to calculate the centre axis of the collimator rotation (CAX) by taking the mean of the centre positions over the sampled collimator rotation range at each gantry angle. The CAX are then projected into the 3D space as lines and the isocentre is determined as a point that minimises the squared summed distance between it and all the projected CAX lines. The centre of the bead is determined from a circular fit to the recognized bead edge points and is used to fix the location of the projected CAXs and their isocentre to the physical space.

The fitting of the field edges is a two-step processes consisting of a coarse and fine-tuning phases. In the coarse step, a rough estimate of the field edge points is obtained by thresholding the portal image at half-value and at 80% of the maximum value to create two masks. The 80% mask is the ran through the convex hull algorithm to remove the possible hole in the mask caused by the attenuation of the bead and the result is merged with the half value mask. The merged mask is then run through the Canny edge detection to find the edge points of the mask, and a function describing a square with the side length and rotation angle as free parameters is fitted to them in polar coordinates.

The fitted square is used to determine its normals and calculate the line profiles of the field edge along them. The profiles are used to determine the field edges more precisely by finding the half value of each profile. A quadrilateral is then fitted to the edge points using the vertices of the fitted square as an initial guess. The fit is performed by connecting the four vertices of the quadrilateral by lines and minimising the summed squared distance between the lines and the edge points simultaneously. A softmin weighting is applied to the distances between the points and lines so that only the edge points between two vertices contribute to the fitting of the line connecting them. After the fitting the field center is calculated from the mean of the determined vertex locations.

The location of the bead is determined in an analogous two-step process where the fitted field edges are used to delineate the area inside which the thresholding is performed to find the rough estimate of the bead edges. A circle with radius and location as the free parameters is fitted to the edges and line profiles are calculated over the edge. The profiles are used to fine tune the edge by selecting the maxima of the gradient as the new edge points. Another circle is then fitted to the fine-tuned edge points, and the centre of the fitted circle is taken as the location of the bead.

To determine the isocentre, the CAX are calculated from the fitted field edge centres by taking their mean over the sampled collimator rotations. The CAX are then projected as lines in 3D in the vicinity of the bead, that is taken to be the (physical) origin of the space. To calculate the direction of the lines, we assume that gantry rotates in the transaxial plane and the nominal gantry angles are accurate enough. The isocentre is then defined to be the point in space that minimizes the summed squared shortest distance to each projected CAX. The size and shape of the isocentre is then calculated from the points of the projected CAX closest to the isocentre.

Technical description of the film analysis

The isocentre and the CAX deviations from it are determined from the starshot pattern exposed to the film as follows. A rough location of the isocentre is determined from the scanned film images by finding the darkest i.e. the most exposed pixel of the image. A circle as large as possible still fitting inside the image is then defined about this point and the angular intensity profile is calculated about its perimeter averaged over a 2 cm wide region. The centre axes of incoming and outgoing beams are then calculated from the profile based on the zero points of the derivative and half values of the beam profiles. The isocentre is then defined as the point closest to the determined beam axes.
